# Exploring Risk Factors Contributing to the Severity of Hazardous Material Transportation Accidents in China

**DOI:** 10.3390/ijerph17041344

**Published:** 2020-02-19

**Authors:** Yingying Xing, Shengdi Chen, Shengxue Zhu, Yi Zhang, Jian Lu

**Affiliations:** 1 College of Transportation Engineering, Tongji University, Key Laboratory of Road and Traffic Engineering of the State Ministry of Education, Shanghai Key Laboratory of Rail Infrastructure Durability and System Safety, Shanghai 201804, China; yingying199004@tongji.edu.cn; 2 School of Transport & Communications, Shanghai Maritime University, 1550 Haigang Street, Shanghai 201306, China; sdchen@shmtu.edu.cn; 3 Jiangsu key Laboratory of Traffic and Transportation Security, Huaiyin Institute of Technology, Huaian 223003, China; zsx10316@hyit.edu.cn; 4 Department of Transportation Engineering, School of Naval Architecture, Ocean & Civil Engineering, Shanghai Jiaotong University, Shanghai 200240, China; darrenzhy@sjtu.edu.cn

**Keywords:** Hazmat, road transportation, accident severity, risk factors, random-parameters ordered probit model

## Abstract

With the increasing demand of hazardous material (Hazmat), traffic accidents occurred frequently during Hazmat transportation, which had caused widespread concern in communities. Therefore, a good understanding of Hazmat transportation accident characteristics and contributing factors is of practical importance. In this study, 1721 Hazmat accidents that have occurred during road transportation for the period 2014–2017 in China were examined, and a random-parameters ordered probit model was established to explore the influence of contributing factors on the severity of accidents by accounting for unobserved heterogeneity in the data. Both the injuries and the number of people evacuated were considered as the indicator of accident severity and investigated, respectively. Results show that higher injury severity is likely to be associated with type of Hazmat (compressed gas, explosive, and poison), misoperation, driver fatigue, speeding, tunnel, slope, county road, dry road surface, winter, dark, more than two vehicles, rear end crash, and explosion. As for the correlation between risk factors and the severity of evacuation, type of Hazmat (compressed gas, explosive, and poison), quantity of Hazmat (10–39 t), misoperation, county road, dry road surface, weekdays, dusk, explosion significantly contribute to increasing the severity of evacuation of Hazmat accidents.

## 1. Introduction

With the continuous improvement of industrialization, the demand of Hazardous material (Hazmat) has been increasing. Road transportation is one of the main ways of Hazmat transportation. According to the Commodity Flow Survey, in 2012, approximately 2.6 billion tons of Hazmat was moved on the U.S. transportation network by all modes. Trucks transported about 60% of these HAZMATs by tonnage [1]. By the end of 2017, approximately 1.1 billion tons of HAZMAT was transported by trucks in China, which accounted for nearly 70% of total Hazmat by all the modes. Besides, hazardous materials in Brazil were also mainly transported by road, accounting for 63% of total Hazmat transportation [2]. To ensure safety of Hazmat transportation, the European Agreement Concerning the International Carriage of Dangerous Goods by Road was developed by the United Nations Economic Commission for Europe [3]. However, frequent road transportation accidents of Hazmat in recent years have shown that Hazmat accidents, which can produce hazards such as fires, explosions, and chemical leaks, not only endanger people’s life, property, and social environment, but also can cause social panic due to the uncertainties of occurrence and consequences. For instance, in 1996, the Class 3 (flammable and combustible liquid) Hazmat incidents caused about $459 million in damage [4]. On 29 March, 2005, a tanker truck containing liquid ammonia collided with a van, causing a large-scale spill of liquid ammonia, resulting in 28 deaths and 350 poisonings, which aroused adverse social impacts. On 19 July, 2014, a truck carrying ethanol and a large bus were involved in a rear-end collision, due to driver fatigue, causing 60 deaths. Compared with other transportation accidents, Hazmat are prone to resulting in more complex problems due to the characteristic of toxicity, corrosiveness, radioactivity, and explosiveness [5]. In recent years, frequent occurrence of accidents caused by Hazmat transportation have resulted in damage to the environment and the catastrophic losses of human life. These accidents have not only attracted increasing concern from the government and the public, but also arouse the attention of scholars, and a growing number of studies about Hazmat transportation accidents were conducted to explore possible countermeasures to improve Hazmat transportation safety [6,7].

Historical analysis of Hazmat accidents is a common method used to reveal their features, such as spatial and temporal distribution, causes, and severity [8]. The researchers largely focused on the collection, analysis, and interpretation of data derived from accident reports or an accident database [7,8,9,10,11,12,13]. For instance, Oggero et al. (2006) analyzed 1932 accidents involving the transportation of Hazmat by road and rail, and found that the majority of the accidents were spill, fires, and explosions and the causes of accidents were weather, driver factors, and mechanical failures [8]. Yang et al. (2010) carried out a study on Hazmat accidents that occurred during the transportation of Hazmat in China. The results indicated that the most frequent types of accident were releases, followed by gas clouds, fires, no substance released due to timely measures, and explosions [11]. Shen (2014) investigated 708 transportation accidents with Hazmat in China from 2004 to 2011 [12]. The results showed that there was a higher probability of large spills in the rollover, run-off, and rear-end collision accidents, and the accidents easily occurred on expressways caused by human errors and vehicle failures. By analyzing 653 surface water pollution accidents in China, Yao et al. (2017) concluded that a high proportion of accidents affected multi-administrative regions and resulted in drinking water crises, and the relevant recommendations for improvement were put out [13]. However, the historical survey of Hazmat accidents is simplistic, and thus, provides limited information about the relationship between risk factors and the severity of Hazmat accidents [14]. 

Some scholars conducted further analysis of Hazmat transportation accidents by using more promising approaches, such as the discrete choice models and Bayesian networks. Chen and Chen (2011) investigated the risk factors affecting injury severity of truck drivers in single- and multi-vehicle crashes on rural highways by using mixed logit model [15]. The results indicated that the likelihood of incapacitating or fatal injury would increase significantly if the truck carries Hazmat, no matter what kind of accident is involved. Zhao et al. (2012) developed a Bayesian belief network to identify the factors associated with Hazmat transportation accidents [16]. They found that the three most important factors that caused Hazmat accidents are human factors, transport vehicle and equipment, the packaging of Hazmat. Zhu et al. (2016) had identified the indispensable factors to result in the Hazmat accidents through Bayesian networks and found that human errors are more likely to result in Hazmat transportation accidents [17]. Uddin and Huynh (2017) investigated the risk factors that influence injury severity of crashes involving Hazmat by using fixed- and random-parameters ordered probit models [14]. Their results showed that male occupants, drivers, crashes occurring in rural locations, under dark-unlighted, under dark-lighted conditions, and on weekdays were more likely to increase the probability of major injuries. Ma et al. (2018) explored the risk factors contributing to Hazmat accidents by using Bayesian network [18]. The results indicated that three or more vehicles involved in Hazmat accidents were more likely to result in severe injury, and the most probable reasons for “explosion accident” were carrying flammable liquids, larger quantity Hazmat, vehicle failure, and transporting in autumn. In addition, the routing and scheduling of Hazmat transportation has also been widely investigated. Different routing optimization models, such as a game-theoretical model [19,20] and a multi-objective model [21,22,23,24], have been designed by considering the impact of different risk factors. Although useful and revealing, these studies paid more attention on the probability or injury severity of Hazmat accidents and provide limited information on the number of people evacuated due to the impact of Hazmat, which is another important and unique measure of Hazmat accident consequences [11].

The purpose of this study is to investigate risk factors that contribute to the severity of Hazmat transportation accidents. Specially, the paper investigates the accidents that occurred during hazmat transportation from 2014 to 2017 in China to identify the risk factors which significantly influence the severity of Hazmat accidents. A random-parameters ordered probit model is developed to account for unobserved heterogeneity in the data. Both the injuries and the number of people evacuated are considered as the indicator of accident severity and investigated, respectively. The results can provide useful reference for Hazmat policy makers and transportation agencies to develop appropriate policies and take necessary measures, thus improving Hazmat transportation safety. 

## 2. Materials and Methods 

### 2.1. Data Source and Study Area

**Figure 1:**
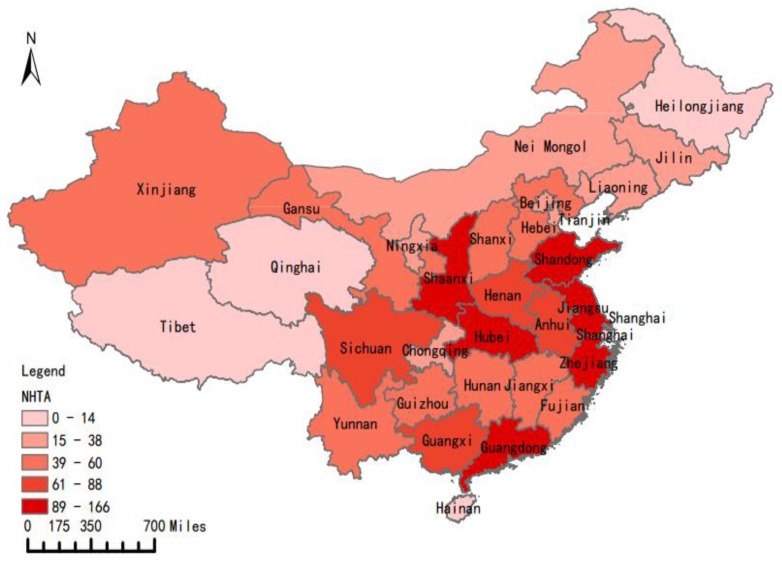
Spatial distribution of Hazmat transportation accidents by provinces.

**Figure 2:**
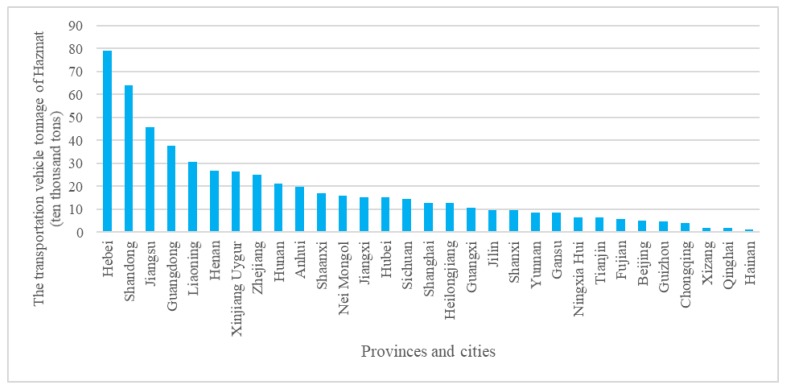
Status of Hazmat road transportation in different provinces at 2016.

The data of Hazmat accidents in the paper was mainly obtained from the State Administration of Work Safety (SAWS), which takes charge of the collection and analysis of all industrial production accidents occurred in China and releases these data to the public regularly [25]. To improve chemical safety and prevent accidents, the National Registration Center for Chemicals (NRCC) of SAWS, has developed a petrochemical accident analysis and data interpretation platform for Hazmat accidents statistics, analysis and interpretation. Besides, the China Chemical Safety Association (CCSA), as an affiliation of SAWS, owns and manages a chemical safety-related database: The Chemical Accident Cases. Combining these two databases, the detailed information of chemical accidents throughout China could be obtained, including numerical (number of injuries and deaths) and qualitative information (substances involved, type of process). This paper focused on the Hazmat accidents during road transport, and accidents that occurred during loading, unloading, storage, and maintenance were excluded. As a result, 1721 Hazmat transportation accidents in China during 2014–2017 were derived from the database. The study area includes 31 provinces and cities in China as the data in Taiwan, Hongkong and Macao are not obtained. Figure 1 shows the number of total Hazmat transportation accidents (NHTA) in different provinces and Figure 2 presents the transportation vehicle tonnage of Hazmat in different provinces in 2016 [26]. It’s obvious that the greater volume of Hazmat transportation by road in more industrialized areas generally resulted in a larger number of Hazmat transportation accidents. For example, Shandong (9.52%), Jiangsu (7.63%), Zhejiang (6.31%) and Guangdong (6.31%) are the provinces with the highest percentages of such accidents, while Tibet, Qinghai, Heilongjiang and Hainan Guizhou, Gansu, Ningxia, and Xinjiang show lower accident frequencies, owing to the smaller scales of chemical industries found in these provinces (as shown in Figure 2). 

Each accident record covers the date, time, location, deaths and casualties, vehicle type, the number of vehicles involved, the quantity and categories of Hazmat, accident type, main causes, and a detailed description of the accident. Thus, risk factors considered in this study were classified into Hazmat factors, driver factors, location factors, environment factors, vehicle factors, and accidents factors. In addition, the information on some risk factors, such as the age and experience of drivers and the vehicle’s speed, were absent in a large number of accident reports; thus, detailed analysis of these factors was not carried out.

### 2.2. Variables

By analyzing the data of Hazmat transportation accidents, the potential factors affecting the severity of accidents are divided into Hazmat factors, driver factors, location factors, environmental factors, vehicle factors and accident factors. And the detailed description of the variables can be found in Table 1.

#### 2.2.1. Hazmat Factors

Hazmat transportation accidents would result in severe consequences due to the characteristics of Hazmat, on the basis of the Hazmat properties, the United Nations classifies Hazmat into nine categories: explosives and pyrotechnics; gasses; flammable and combustible liquids; flammable, combustible, and dangerous-when-wet solids; oxidizers and organic peroxides; poisonous and infectious materials; radioactive materials; corrosive materials; and miscellaneous dangerous goods [27]. Based on the Hazmat involved in accidents, the Hazmat in this study is classified into 8 categories: explosives; gasses; flammable liquids; flammable solids; oxidizers and organic peroxides; poisons; corrosives; and others. In addition, according to previous study, the quantity of Hazmat is divided into 4 groups: <10, 10–24, 25–39, and ≥40 tons [18].

#### 2.2.2. Driver Factors

The behavior of drivers is considered as the potential risk factors. Certain risky driving behaviors could increase the probability of accidents, such as misoperation, speeding, and driver fatigue. 

#### 2.2.3. Location Factors

The road types are classified into five groups according to their functions, i.e., county road, provincial road, state road, city road and freeway. And road surface condition is classified as dry or wet [14]. Special locations may be associated with the probability of accidents, such as tunnel, curve, slope, and ramp.

#### 2.2.4. Environment Factors

Seasons could be divided into: spring (March–May), summer (June–August), autumn (September–November) and winter (December–February) [28]. Weekly distribution of accidents is defined as weekends and weekdays (Monday–Friday). In addition, the holiday is also considered as the risk factor. Lighting intensity is divided into: dawn (5:00–6:59 a.mm), daytime (7:00 a.m.–4:59 p.m.), dusk (5:00 p.m.–6:59 p.mm), dark (7:00 p.m.–4:59 a.m.) [16]. Weather is divided into: sunny or cloudy, and rainy, fog, or snow.

#### 2.2.5. Vehicle Factors

Three major vehicle types are considered in the paper: private car, bus, truck, as well as the non-motor, and the accident records indicate that the accident severity could be affected by the mixture of car, bus and truck. Number of vehicles involved in accidents is divided into 4 categories: one, two, three, and more than three vehicles.

#### 2.2.6. Accident Factors

The type of accident can be divided into: rear-end, sideswipe, rollover, head on collision, vehicle failure (including brake failure, tire blowout, spontaneous combustion, and tanker damage). The consequences of Hazmat transportation accidents fall into four categories: explosion, fire, spill, and non-spill.

### 2.3. Definition of Accidents Severity

The severity of accidents is usually divided into five categories [14,29]: no injury, possible injury (or minimal injury), non-incapacitating injury (or evident injury, minor injury), disabling injury (or incapacitating injury, major injury), and fatality. However, due to the relevance of the different categories, the severity of accident was divided it into four categories, which could ease the issue of potential relationship of related accident consequence, and ensure the sufficient sample size for the model [15,30].

No injury (level 1): the people involved in accidents sustained no injuries at all.
Minor injury (level 2): the victim need not to be admitted to the hospital.
Severe injury (level 3): the victim was admitted to the hospital either for treatment or observation.
Fatality (level 4): the victim died within 30 days of collision or on site.

**Figure 3:**
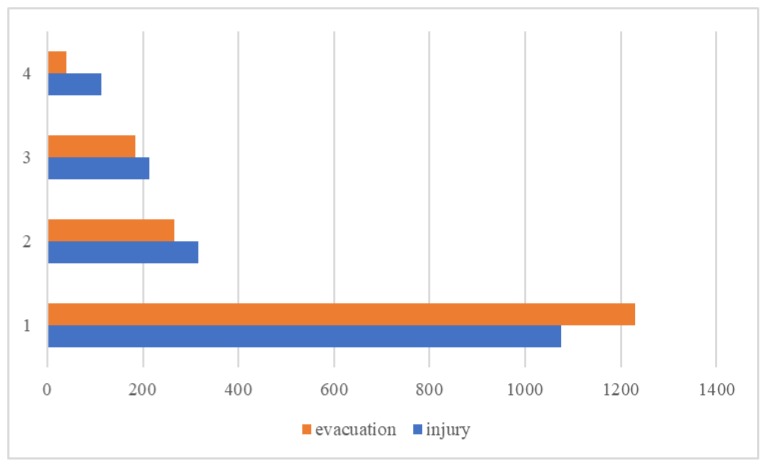
Frequency of reported Hazmat transportation accidents in each injury and evacuation severity category.

In addition, considering the characteristic of Hazmat, the severity of evacuation also should be taken into account and is divided into four categories according to the number of people evacuated: no evacuation (level 1); minor evacuation (level 2, the evacuation of 1–100 people); general evacuation (level 3, the evacuation of 101–1000 people); and severe evacuation (level 4, more than 1000 people) [11]. Figure 3 shows the reported number of Hazmat transportation accidents in each injury and evacuation severity category.

### 2.4. Methods

Discrete choice models, such as ordered probit/logit models and nested logit models, have been extensively used in exploring the relationship between risk factors and injury severity in the traffic safety field [31,32,33]. In the study, there are 20 independent variables, and the dependent variable is the injury severity, and the severity of the evacuation, which are ordered variables. As a result, the ordered-response models were used to investigate the risk factors that contribute to the severity of injuries and evacuation in Hazmat accidents. As the ordered-probit model follows a normal distribution and does not suffer from estimation difficulties associated with the multinomial probit model, the ordered-probit model is typically selected in preference to the ordered logit model [33]. Ordered probit (OP) models are built around a latent regression [34]:

(1) $$y^{*}=\beta X+\varepsilon$$

where $y^{*}$ is the continuity latent variable, and could be expressed as $y^{*}=\beta X+\varepsilon$; β is the vector of estimable parameters; X is the vector of explanatory variables; and ε is the error term, which is assumed to be normally distributed with zero mean and unit variance.

Then, the observed data y can be determined from the model as follows:

(2) $$y_{i}=\{\begin{array}{c} 1\;if\;y_{i}^{*}\leq c_{1}\\ 2\;if\;c_{1}<y_{i}^{*}\leq c_{2}\\ 3\;if\;c_{2}<y_{i}^{*}\leq c_{3}\\ 4\;if\;c_{3}<y_{i}^{*} \end{array}$$

where $y_{i}$ is considered as the severity of injuries or evacuation in Hazmat accidents. Taking injury severity level as an example, $y_{1}$ expresses as no injury, $y_{2}$ is the minor injury, $y_{3}$ is the severe injury, and $y_{4}$ is considered as fatality. c is the estimable parameter (referred to as threshold) that is estimated jointly with β.

To calculate the probabilities of injury and evacuation severities for a given X, the y could be calculated by $y^{*}$ parameters. And the probabilities could be expressed as:

(3) $$Pr(y=i|X)=\{\begin{array}{cc} \varphi \left(c_{1}-\beta X\right) & if\;i=1\\ \varphi \left(c_{2}-\beta X\right)-\varphi \left(c_{1}-\beta X\right) & if\;i=2\\ \varphi \left(c_{3}-\beta X\right)-\varphi \left(c_{2}-\beta X\right) & if\;i=3\\ 1-\varphi \left(c_{3}-\beta X\right) & if\;i=4 \end{array}$$

where φ() is the standard normal cumulative distribution function.

In addition, to allow for the effect of the variables to vary across observations and capture the unobserved heterogeneity in the data, a random-parameters ordered probit model was developed by considering [28]:

(4) $$\beta_{i}=\beta +\mu_{i}$$

where $\beta_{i}$ is a vector of household-specific parameters, $\mu_{i}$ is the randomly-distributed term.

A practical issue in this model is that its estimated coefficients are not sufficient to clarify the direction and magnitude of interior categories (i.e., severe evacuation in this analysis). Therefore, marginal effects of the parameters are often used to reveal the direction of the effects of the parameters on these categories. The marginal effects of factors *X* on the severity of injury and evacuation can be calculated in the following way:

(5) $$\frac{\vartheta Pr(y=i)}{\vartheta X}=\{\begin{array}{cc} \varphi \left(c_{1}-\beta X\right)\beta {}^{\prime } & if\;i=1\\ {}[\varphi \left(c_{1}-\beta X\right)-\varphi \left(c_{2}-\beta X\right)]\beta {}^{\prime } & if\;i=2\\ \left[\varphi \left(c_{2}-\beta X\right)-\varphi \left(c_{3}-\beta X\right)\right]\beta {}^{\prime } & if\;i=3\\ \varphi \left(c_{3}-\beta X\right)\beta {}^{\prime } & if\;i=4 \end{array}$$

where $\beta {}^{\prime }$ denotes the effect of changes in the explanatory variables on the underlying scale.

## 3. Results and Discussion

The random-parameters ordered probit specifications were estimated using the simulated maximum likelihood method, and the statistical software NLOGIT 6.0 (Econometric Software, Inc.: Plainview, NY, USA) was used for model estimation [34]. In the simulation process, Halton draws was used to maximize the simulated likelihood function as previous studies had demonstrated that Halton draws could produce a more efficient distribution of draws for numerical integration than purely random draws [35,36,37]. The density functions of the random parameters can follow many distributions, such as normal, log-normal, triangular, and uniform. For this study, all these distributions were tested and the normal distribution was found to provide the best statistical fit, which is consistent with previous research [28,38,39]. If the estimated standard deviations were significantly different from zero (at 90% level), the parameters were identified as random parameters, otherwise, as fixed parameters across the accidents. Model goodness-of-fit was tested and compared using the Akaike Information Criterion (AIC), Bayesian Information Criterions (BIC) and $R^{2}$, which are the most used fit statistics displayed in statistical model output. A smaller AIC indicates a better fitted model, it is generally formulated as:

(6) AIC=−2L+2k

where L is the model log-likelihood, *k* is the number of predictors including the constant.

The *BIC* is calculated by the following equation,

(7) BIC=−2L+ln(n)k

where L is the model log-likelihood, *k* is the number of predictors including the constant, n is the sample size.

Table 2 presents fit statistics of the random-parameters ordered probit (RPOP) model (using 200 simulated Halton draws) and fixed-parameters ordered probit model (FPOP), respectively. Given both RPOP and FPOP models fit on the same dataset, the model with lower AIC and BIC, and higher $R^{2}$ is considered to outperform the other one [14]. Table 2 suggests that the random parameters model has lower AIC (2821.42 vs. 2941.22; 2119.94 vs. 2213.14), lower BIC (3320.88 vs. 3440.68; 2619.40 vs. 2712.60), and higher R2 (0.12 vs. 0.08; 0.17 vs. 0.13). Therefore, the random-parameters model has a better fit than the fixed-parameters model. In addition, the values of $R^{2}$ are relatively low because many variables (e.g., drive age, driver experience, and traffic signs) are not measured [40]. Table 3 shows the estimated results of RPOP models and Table 4 lists and marginal effects of the explanatory variables on different severity levels of injury and evacuation. It should be noted that the only risk factors that are significantly associated with severity levels of injury and evacuation were showed in Table 3 and Table 4.

From Table 3, all the factors but quantity of Hazmat, road types, ramp, curve, weekly distribution, holidays, weather, type of accidents are statistically significant predictors (at 90% level) for the model. Four variables: type of Hazmat (compressed gas, poison, and corrosives), misoperation, wet road surface, accident consequence (spill, non-spill), were found to be random parameters (all normally distributed), suggesting that their effect varies across the observations. As for the correlation between risk factors and the severity of evacuation, eight variables were found to be statistically significant at 90% confidence interval, i.e., type of Hazmat, quantity of Hazmat, misoperation, freeway, road surface, weekly distribution, lighting intensity, accident consequence, and five variables: type of Hazmat (compressed gas, poison, and corrosives), misoperation, freeway, wet road surface, and accident consequence (spill, non-spill), were found to be random. Then, these significant risk factors were discussed in detail below.

### 3.1. Hazmat Factors

The explosive is identified as a fixed parameter that is significantly associated with the severity of injury and evacuation of Hazmat accidents, and according to the obtained results, explosive increases the likelihood of minor, severe and fatal injury by 0.007, 0.016, and 0.006, and increases the likelihood of minor, general and severe evacuation by 0.052, 0.030, and 0.009, respectively. This result is consistent with the previous finding that accidents involving explosives are more severe than those involving flammable substances [41]. The main reason is that the explosives could cause serious consequences, such as fires and explosions due to its characteristics, posing a great threat to occupants, ecological environment, and surrounding buildings. Gases and poisons are both found as significant factors for the severity of injuries and evacuation in Hazmat accidents. Besides, the parameters for compressed gas and poison estimates are found to be random across the observed crashes, indicating that their estimated standard deviations are statistically significantly different from zero. Compared with flammable liquids, gases and poisons are not only associated more with minor, severe, and fatal injuries, but also increase the probabilities of minor, general, and severe evacuation. This is probably due to the diffusivity, explosivity of gases, and toxicity of poisons, which may affect the health of more people and cause significant environment impact. According to the statistics, if the flammable gas leaks, the probability of fire is 19.5% and the probability of explosion after a fire is 75%, while the probability of fire after flammable liquid leakage is 39.6%, and the probability of explosion after a fire is 47.8%. This indicates that the flammable liquid leaks more easily than the flammable gas, but it is much less likely to turn into an explosion than the flammable gas [32]. Corrosives are found to be a random parameter that has a negative effect on the probability of severe-injury and severe-evacuation accidents, and corrosives could increase the likelihood of no injury by 0.056, and could increase the likelihood of no evacuation by 0.061, respectively. This result is also in line with the study conducted by Wu et al. (2014) [42]. They found that less casualties are caused by the spill of corrosives.

As for quantity of Hazmat, it has positive impact on the probability of severe-evacuation accidents, and the likelihood of minor, general, and severe evacuation increased by 0.019, 0.010, and 0.002 when the truck load is between 25 and 39 tons. Similarly, compared with the quantity of less than 10 tons, the likelihood of minor, general and severe evacuation increased by 0.025, 0.017, and 0.006 when the truck load is more than 40 tons. These could be explained by that the larger amount of Hazmat transportation, the larger inertia of the transportation vehicles, making it difficult for the driver to steer when an emergency happens and increasing the probability and severity of a crash [18,43]. Moreover, in case of spill, fire and explosion, the larger amount of Hazmat is more prone to result in serious consequences, threating people’s health and environment.

### 3.2. Driver Factors

Many studies have shown that driver’s driving status significantly affects the severity of Hazmat transportation accidents [44,45]. Misoperation is identified as a random parameter that significantly contributed to the probability of severe-injury and severe-evacuation accidents. It could increase the likelihood of minor, severe and fatal injuries by 0.024, 0.062, and 0.026, and the likelihood of minor, general and severe-evacuation by 0.005, 0.003, and 0.001, respectively. 

Driver fatigue and speeding are both found to be fixed parameters that significantly influence the injury severity of Hazmat accidents. Based on the result, driver fatigue increases the likelihood of minor, severe, and fatality-injury by 0.042, 0.105, and 0.045, while speeding is 0.022, 0.055, and 0.023. The results are in agreement with previous studies [45,46,47,48]. Analyzing the accident data, driver fatigue usually occurs from midnight to earlier morning, which is a period of physical inactivity and exhaustion of a driver and easily leads to driver fatigue. 

### 3.3. Location Factors

**Figure 4:**
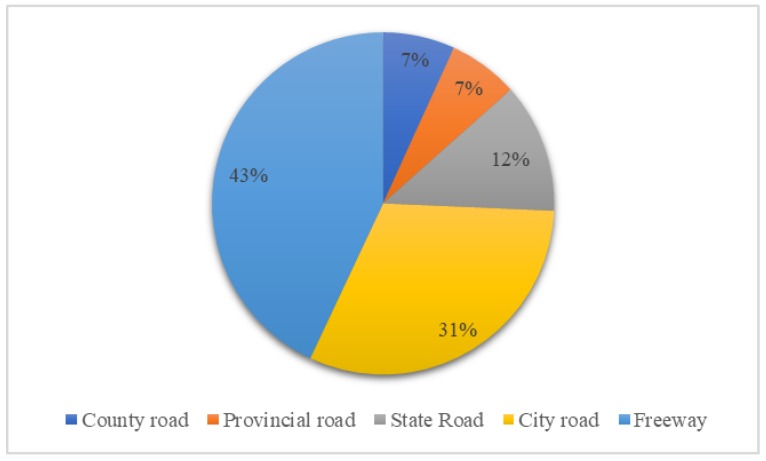
The distribution of Hazmat transportation accidents by road types.

**Figure 5:**
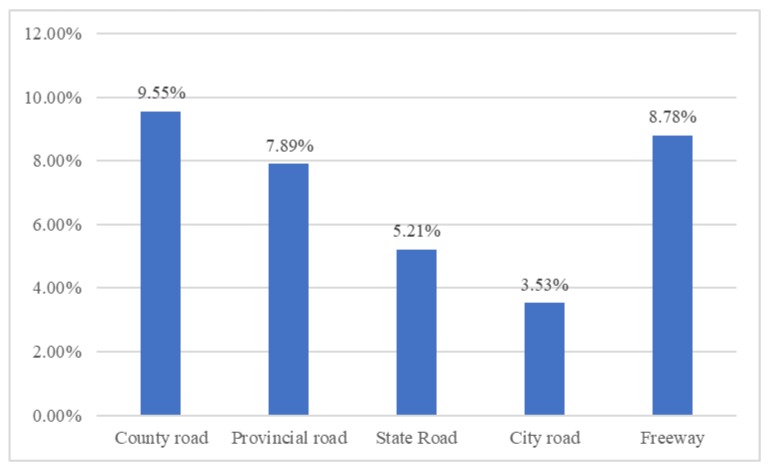
The proportion of fatal injury of total number of Hazmat accidents by road types.

With respect to accidents that occurred on a freeway, the results indicate that it is a random parameter as its estimated standard deviation is statistically significantly different from zero. Based on Table 3, the likelihood of no injury increases by 0.029 and the likelihood of no evacuation increases by 0.059 when the Hazmat accidents occurs on freeway. The main reason is that the county roads are mainly undivided rural roads in small cities in China, which usually result in higher injury severity [14,49]. Although more than 40% (as shown in Figure 4) of the Hazmat accidents occur on the freeway, the probability of fatal accidents is the highest (as shown in Figure 5) on county roads due to their poor driving and rescue conditions, resulting in the highest percentage of fatal accidents and general evacuation. Besides, according to Annul Development Report of Chinese Hazardous Chemicals Industry (2017), many counties and villages located near the county roads bring unpredictable safety risk to nearby residents [50]. 

Hazmat accidents occurring on wet road are found to be less severe than that on dry road. This variable is also found to be a random parameter with statistically significant standard deviation. The results show that wet surface conditions are associated with increase of 0.044 and 0.067 in probabilities of no injury and no evacuation, respectively. This is probably because drivers tend to be more cautious while driving on wet surfaces and reduce their speed [39,51,52]. The effect of weather in lieu of road-surface condition is also examined and found to be insignificant. 

Accidents occurred at the tunnel are found to be a fixed parameter, and is associated with increased likelihood of minor, severe, and fatal injuries, increasing the likelihood by 0.038, 0.163, and 0.132, respectively. Although there are only a few Hazmat accidents occurring inside tunnels, they usually lead to enormous damage to the infrastructure and people in the vicinity of the accident as it is difficult to escape from the tunnel and to perform rescues [8,12]. When a Hazmat accident occurred on a slope, occupants are more likely to sustain severe injuries. To be specific, the occupants are associated with an increase in the risk of minor, severe, and fatal injuries by 0.029, 0.096, and 0.058, respectively. This result is in line with the findings of Duncan et al. (1998) and Chen and Chen (2011) [15,31]. As reported by Chang (2005), grade has a significant effect on vehicle operation speed, particularly for large trucks and buses [53]. Moreover, Fu et al. (2011) pointed out that driving on continuous descending roads may lead to braking problems to HGVs (Heavy Goods Vehicles) [54]. Then, vehicle speed becomes out of control and accidents are prone to follow due to serious brake failure. 

### 3.4. Environment Factors

**Figure 6:**
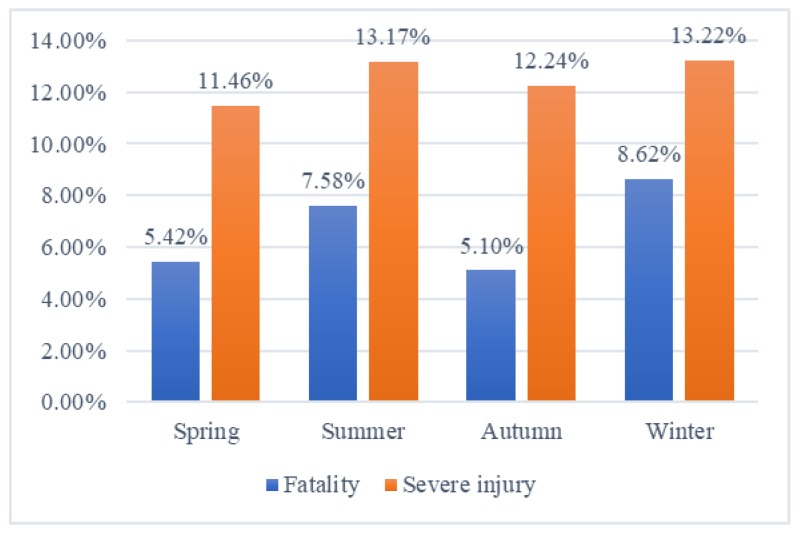
The proportion of fatal and severe injury of total number of Hazmat accidents by seasons.

The autumn and spring are found to be fixed parameters that has significant and negative effect on the probability of severe-injury accidents. Autumn decreases the likelihood of minor, severe, and fatal injuries by 0.013, 0.031, and 0.013, and spring is 0.015, 0.035, and 0.014, respectively. This is probably because foggy and snowy weather conditions typically occur in the colder months of late autumn and winter [55]. Although, the summer is not statistically significant in this model, it still has a great influence on the injury severity. As shown in Figure 6, the proportion of fatal and severe injury is highest in the winter, followed by the summer. This is probably due to the solarization and high temperature in summer, which may cause the spontaneous combustion of some flammable and combustible materials. Previous studies also show that the summer months tend to increase the possibility of injury severity [56,57,58].

Weekdays are a fixed parameter that have a positive effect on the probability of severe-evacuation accidents, this could be ascribed that roadways carry higher volumes of traffic during weekdays. This finding is in line with the finding of Islam and Hernandez (2013a) and Uddin and Huynh (2017) [14,59].

As for the lighting intensity, dusk is found to be fixed parameter that has a negative effect on the probability of severe-injury accidents. Dusk (5:00 p.m.–6:59 p.m.) would decrease the likelihood of minor, severe, and fatality accidents by 0.026, 0.057, and 0.02 than daytime (7:00 a.m.–4:59 p.m.). This is because most transportation corporations are more likely to transport Hazmat at night in China [60]. In addition, poor visibility at night would make drivers tired, which would result in driver fatigue, especially during 11:00 p.m.–3:00 a.m. [61]. In the data sample, 62% of total accidents are caused by driver fatigue during the period from 7:00 p.m. to 4:59 a.m. In contrast with the negative effect of dusk on the injury severity of Hazmat accidents, it would increase the likelihood of minor, general, severe evacuation by 0.049, 0.028, and 0.008. This is probably due to lower traffic volumes at night.

### 3.5. Vehicle Factors

Among vehicle factors considered, the results show that the relationship between injury severity and number of vehicles involved in Hazmat accidents is statistically significant. More than three vehicles and three vehicles have positive effect on the level of injury, and the likelihood of minor, severe, and fatal injuries increased by 0.033, 0.117, and 0.076 when three vehicles involved in accidents, while more than three vehicles increased the likelihood of minor, severe, and fatal injuries by 0.034, 0.121, and 0.080, respectively. The results are consistent with the findings of Laman (2012) and Shinstine et al. (2016) [62,63].

### 3.6. Accident Factors

Many studies have shown that the significant relationship of accidents type and the severity of injury, indicating that the rear-end is more likely to lead to severe injuries [31,32,64,65,66]. Our model results show that sideswipe and vehicle failure have negative effect on the severe injury accidents in comparison with rear-end and sideswipe would decrease the likelihood of minor, severe, and fatal accidents by 0.045, 0.089, and 0.027, and vehicle failure is 0.048, 0.104, and 0.037, respectively. 

**Figure 7:**
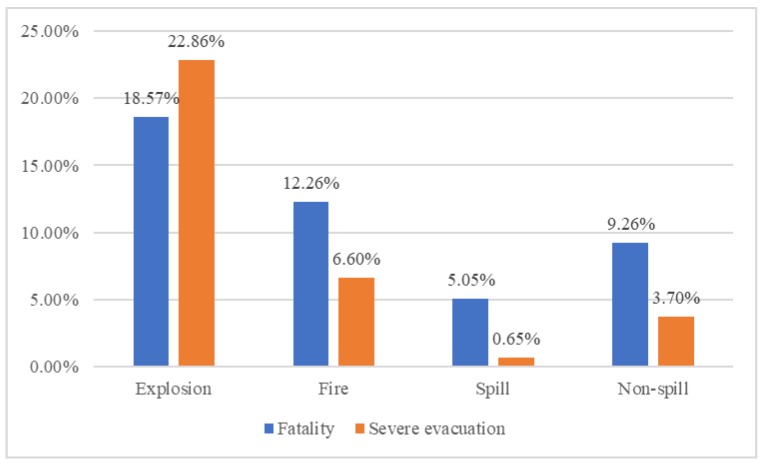
The proportion of fatality and severe evacuation of total number of Hazmat accidents by accident consequences.

Compared with explosion, fire is a fixed parameter, which has shown the negative effect on the likelihood of severe-injury and evacuation accidents, and it would decrease the likelihood of minor, severe and fatality-injury by 0.001, 0.090, and 0.031, and could decrease the likelihood of minor, general, and severe-evacuation by 0.073, 0.03, and 0.007, respectively. Spill, and non-spill are found to be random parameters and significantly affect the severity of injury and evacuation in Hazmat accidents. The results show that compared to an explosion, spill, and non-spill are both associated with a decrease in the probability of severe injuries and evacuation. The main reason is that explosion caused by Hazmat accidents would lead to severe consequences due to its characteristic, especially in the urban area and higher population densities [67]. This finding could be explained by the nature of explosion. Explosion could instantaneously discharge enormous energy with thermal radiation and debris impact, which could be difficult for people to escape quickly [60]. Therefore, the explosion results in the highest number of fatal injuries and severe evacuations (as shown in Figure 7).

## 4. Conclusions and Recommendation

This study focuses on the risk factors that affect the safety of Hazmat transportation. All Hazmat transportation accidents (*N* = 1721) occurred in China during 2014 to 2017 are considered. The potential risk factors retrieving from database mainly include Hazmat, driver, location, environment, vehicle, and accident factors. The importance of different risk factors in the Hazmat transportation accidents are analyzed by a random-parameter ordered probit model, and the impact of the potential risk factors on accidents severity could be obtained. 

Results show that higher severity of the injury and the evacuation are likely to be associated with type of Hazmat, namely compressed gas, explosive, and poison. Among these three Hazmats, the explosive leads to the most serious injury, which increase the likelihood of minor, severe and fatal injury by 0.007, 0.016, and 0.006, respectively, while the compressed gas causes more people to be evacuated than other types. Therefore, the transportation corporation should avoid densely populated areas when transporting Hazmat, especially the explosive and the compressed gas. The quantity of Hazmat that the truck transports has positive impact on the probability of severe-evacuation accidents, and the likelihood of minor, general, and severe evacuation increased by 0.024, 0.012, and 0.003, respectively when the truck load increases from less than 10 tons to between 10 and 39 tons. 

Drivers’ driving status is closely related with the Hazmat accidents. Driver’s misoperation, driver fatigue and speeding are more likely to increase the probability of severe-injury. Among these three risky behaviors, the highest increase of injury severity is caused by the driver fatigue. In addition, the misoperation of drivers also leads to the increase of likelihood of severe evacuation. Therefore, Hazmat transportation corporations should be strict with the drivers’ skill and operation, and the education programs on safe transportation of Hazmat for drivers should be carried out to decrease the risky driving (such as: driver fatigue, speeding) by the Hazmat transportation corporations. Moreover, the fatigue detection system could be installed to monitor the driver’s posture to recognize drowsiness. 

Road types, road surface conditions, and special locations are potential risk factors affecting Hazmat transportation safety. Hazmat transportation on county roads, dry road surface and special sections, i.e., tunnel and slope, are significantly contributing to increasing the injury severity of Hazmat accidents, while Hazmat transportation on county roads and dry road surface also have a positive effect on the severity of evacuation. This implies that the tunnel and the long and steep slope should be avoided as much as possible when selecting route for Hazmat transportation. Otherwise, the traffic management department should enhance the supervision and management of these dangerous locations and establish an effective response system. 

Compared with the winter, the autumn and spring have lower probability of severe-injury accidents. As for the lighting intensity, the dark is more likely to increase the probability of severe-injury accidents, while the dusk significantly contributes to increasing the severity of evacuation of Hazmat accidents. Weekdays are also found to have a positive effect on the probability of severe-evacuation accidents.

Among vehicle factors considered, the number of vehicles has significant effect on the injury severity, while vehicle types do not show an obvious correlation. More than two vehicles involved in Hazmat accidents tend to increase the likelihood of severe-injury and fatal injury. It may be considered to separate Hazmat vehicles with other vehicles spatially and temporally, for example, Hazmat vehicles may only be allowed to transport during a specific time period or on a dedicated lane.

In comparison with other accident types, rear-end collision are more likely to lead to severe-injury and severe-evacuation accidents. To reduce the severity of accidents, drivers’ safety awareness and adaptability should be improved to enhance their vehicle control capabilities and keep a safe distance from the vehicle in front, enough for putting on the emergency brake.

With regard to accident consequence, explosion would result in the most serious consequence and increase the likelihood of severe-injury and evacuation accidents. Therefore, once a Hazmat accident occurs and leaks, drivers should ensure their own safety first. Then, all fire ignition sources, which may set off an explosion, should be eliminated in the hazardous area immediately. In addition, drivers should master the necessary emergency measures for Hazmat accidents, which could help to reduce the risk of secondary accidents.

## Figures and Tables

**Table 1:** Frequency distribution of Hazmat road transportation accidents.

Factors	Variables	Description	Count (Proportion)
Hazmat factors	Type of Hazmat	Explosives	24 (1.4%)
		Gases	248 (14.4%)
		Flammable liquids	910 (52.9%)
		Flammable solids	26 (1.5%)
		Oxidizers and organic peroxides	13 (0.8%)
		Poison	57 (3.3%)
		Corrosives	245 (14.2%)
		Others	198 (11.5%)
	Quantity of Hazmat	<10 tons	526 (30.6%)
		10–24 tons	436 (25.3%)
		24–39	641 (37.2%)
		≥40 tons	118 (6.9%)
Driver factors	Misoperation	No	1593 (92.6%)
		Yes	128 (7.4%)
	Driver fatigue	No	1678 (97.5%)
		Yes	43 (2.5%)
	Speeding	No	1652 (96.0%)
		Yes	69 (4.0%)
Location factors	Road types	County road	117 (6.8%)
		Provincial road	114 (6.6%)
		State Road	211 (12.3%)
		City road	539 (31.3%)
		Freeway	740 (43.0%)
	Road surface	Dry	1380 (80.2%)
		Wet	341 (19.8%)
	Tunnel	No	1648 (95.8%)
		Yes	73 (4.2%)
	Ramp	No	1687 (98%)
		Yes	34 (2.0%)
	Curve	No	1655 (96.2%)
		Yes	66 (3.8%)
	Slope	No	1664 (96.7%)
		Yes	57 (3.3%)
Environment factors	Seasons	Winter	480 (27.9%)
		Summer	501 (29.1%)
		Autumn	392 (22.8%)
		Spring	348 (20.2%)
	Weekly distribution	Weekend	1314 (76.4%)
		Weekday	407 (23.6%)
	Accidents occurred at holidays	No	1660 (96.5%)
		Yes	61 (3.5%)
	Lighting intensity	Dark	524 (30.4%)
		Daytime	874 (50.8%)
		Dusk	124 (7.2%)
		Dawn	199 (11.6%)
	Weather	Sunny	424 (24.6%)
		Cloudy	747 (43.4%)
		Rainy, fog and snow	550 (32.0%)
Vehicle factors	Number of vehicles	1	1028 (59.7%)
		2	571 (33.2%)
		3	60 (3.5%)
		≥4	62 (3.6%)
	Vehicle types	Hazardous trucks	961 (55.8%)
		Hazardous truck and truck	586 (34.0%)
		Private car & Truck	110 (6.4%)
		Non-motor & Truck	29 (1.7%)
		Bus & Private car & Truck	11 (0.6%)
		Bus & Truck	24 (1.39%)
Accidents factors	Type of accidents	Rear-end	395 (23.0%)
		Sideswipe	31 (1.8%)
		Rollover	636 (37.06%)
		Head on collision	295 (17.1%)
		Vehicle failure	364 (21.2%)
	Accident consequence	Explosion	55 (3.2%)
		Fire	204 (11.9%)
		Spill	1170 (68.0%)
		Non-spill	292 (17.0%)

**Table 2:** Comparison of fit statistics of random-parameters ordered probit (RPOP) and fixed-parameters ordered probit model (FPOP) models.

Model Fit Statistics	RPOP (Injury)	FPOP (Injury)	RPOP (Evacuation)	FPOP (Evacuation)
Log likelihood	−1317.71	−1377.61	−966.97	−1013.57
AIC	2821.42	2941.22	2119.94	2213.14
BIC	3320.88	3440.68	2619.40	2712.60
Pseudo R-square	0.12	0.08	0.17	0.13

**Table 3:** Estimated results of the RPOP model.

		Injury		Evacuation	
		Parameter Estimate	z-Statics	Parameter Estimate	z−Statics
	Constant	−1.26 **	−2.49	−1.13 *	−1.95
	Threshold u_1_	0.28 ***	10.66	0.68 ***	12.68
	Threshold u_2_	1.12 ***	17.04	1.43 ***	12.62
Hazmat factors	**Type of Hazmat (Reference: Flammable Liquids)**				
	Gases	0.02 (0.40 ***)	0.22 (3.45)	0.47 *** (0.26 **)	4.71 (2.41)
	Explosives	1.39 ***	4.07	1.66 ***	6.68
	Poisons	0.03 (0.44 *)	0.16 (1.95)	0.32 *(0.72 ***)	1.6 (2.65)
	Corrosives	−0.18 * (0.34 ***)	−1.68 (3.13)	−0.13 (0.72 ***)	−1.09 (4.74)
	Others	−0.29 **	−2.05	−−−	−−−
	**Quantity of Hazmat (Reference:<10)**				
	25−39	−−−	−−−	0.15 **	2.01
	≥40	−−−	−−−	0.23 **	2.45
Driver factors	**Misoperation (Reference: No)**				
	Yes	0.37 ***(0.25 ***)	3.00 (7.61)	0.05 (0.07 **)	0.32 (2.21)
	**Driver fatigue (Reference: No)**				
	Yes	0.63 ***	2.91	−−−	−−−
	**Speeding (Reference: No)**				
	Yes	0.33 *	1.69	−−−	−−−
Location factors	**Road Types (Reference: County Road)**				
	Provincial road	−−−	−−−	−−−	−−−
	State Road	−−−	−−−	−−−	−−−
	City road	−−−	−−−	−−−	−−−
	Freeway	−0.04 (1.22 ***)	0.27 (7.18)	−0.32 ** (0.66 ***)	2.08 (4.02)
	**Road Surface (Reference: Dry)**				
	Wet	−0.20 (0.18 **)	1.85 (2.07)	−0.31 *** (0.38 ***)	2.67 (3.74)
	**Tunnel (Reference: No)**				
	Yes	0.91 ***	3.89	−−−	−−−
	**Grade (Reference: No)**				
	Yes	0.52 ***	2.82	−−−	−−−
Environment factors	**Seasons (Reference: Winter)**				
	Autumn	−0.20 *	−1.74	−−−	−−−
	Spring	−0.22 **	−2.08	−−−	−−−
	**Weekly Distribution (Reference: Weekends)**				
	Weekdays	−−−	−−−	0.14 *	1.53
	**Lighting Intensity (Reference: Dark)**				
	Dusk	−0.39 **	−2.22	0.28 **	1.97
Vehicle factors	**Number of Vehicles (Reference:1)**				
	3	0.64 ***	2.93	−−−	−−−
	≥4	0.66 ***	2.90	−−−	−−−
Accidents factors	**Type of Accidents (Reference: Rear−End)**				
	Sideswipe	−0.74 *	−1.94	−−−	−−−
	Vehicle failure	−0.76 ***	−3.81	−−−	−−−
	**Accident Consequence (Reference: Explosion)**				
	Fire	−0.68 ***	−3.31	−0.49 ***	−2.63
	Spill	−0.87 *** (0.88 ***)	−4.90 (6.32)	−0.64 *** (0.58 ***)	3.90 * (2.91)
	Non−spill	−0.68 *** (0.49 **)	−2.61 (2.1)	−0.74 *** (1.50 ***)	−2.66 (8.33)

Note 1: ***, **, *, respectively, mean 99%, 95%, and 90% level of confidence. Note 2: u_0_ is normalized to zero.

**Table 4:** Marginal effects of the explanatory variables.

		Injury				Evacuation			
		No Injury	Minor Injury	Severe Injury	Fatality	No Evacuation	Minor Evacuation	General Evacuation	Severe Evacuation
**Type of Hazmat (Reference: Flammable Liquids)**									
Hazmat factors	Gases	−0.022	0.005	0.012	0.005	−0.143	0.080	0.048	0.015
	Explosives	−0.511	−0.007	0.016	0.006	−0.091	0.052	0.030	0.009
	Poisons	−0.019	0.004	0.010	0.004	−0.043	0.025	0.013	0.004
	Corrosives	0.056	−0.011	−0.031	−0.014	0.061	−0.039	−0.017	−0.004
	Others	0.081	−0.019	−0.045	−0.017	−−−	−−−	−−−	−−−
	**Quantity of Hazmat (Reference: <10 tons)**								
	25−39	−−−	−−−	−−−	−−−	−0.035	0.019	0.010	0.002
	≥40	−−−	−−−	−−−	−−−	−0.056	0.025	0.017	0.006
Driver factors	**Misoperation (Reference: No)**								
	Yes	−0.113	0.024	0.062	0.026	−0.838	0.005	0.003	0.001
	**Driver fatigue (Reference: No)**								
	Yes	−0.191	0.042	0.105	0.045	−−−	−−−	−−−	−−−
	**Speeding (Reference: No)**								
	Yes	−0.100	0.022	0.055	0.023	−−−	−−−	−−−	−−−
Location factors	**Road Types (Reference: County Road)**								
	Provincial road	−−−	−−−	−−−	−−−	−−−	−−−	−−−	−−−
	State Road	−−−	−−−	−−−	−−−	−−−	−−−	−−−	−−−
	City road	−−−	−−−	−−−	−−−	−−−	−−−	−−−	−−−
	Freeway	0.029	−0.018	−0.009	−0.022	0.059	−0.037	−0.018	−0.004
	**Road Surface (Reference: Dry)**								
	Wet	0.044	−0.010	−0.024	−0.010	0.067	−0.042	−0.020	−0.005
	**Tunnel (Reference: No)**								
	Yes	−0.332	0.038	0.163	0.132	−−−	−−−	−−−	−−−
	**Slope (Reference: No)**								
	Yes	−0.183	0.029	0.096	0.058	−−−	−−−	−−−	−−−
Environment factors	**Seasons (Reference: Winter)**								
	Autumn	0.057	−0.013	−0.031	−0.013	−−−	−−−	−−−	−−−
	Spring	0.064	−0.015	−0.035	−0.014	−−−	−−−	−−−	−−−
	**Weekly Distribution (Reference: Weekends)**								
	Weekdays	−−−	−−−	−−−	−−−	−0.850	0.088	0.044	0.019
	**Lighting Intensity (Reference: Dark)**								
	Dusk	0.004	−0.026	−0.057	−0.020	−0.015	0.049	0.028	0.008
Vehicle factors	**Number of Vehicles (Reference:1)**								
	3	−0.020	0.033	0.117	0.076	−−−	−−−	−−−	−−−
	≥4	−0.234	0.034	0.121	0.080	−−−	−−−	−−−	−−−
Accidents factors	**Type of Accidents (Reference: Rear−End)**								
	Sideswipe	0.045	−0.045	−0.089	−0.027	−−−	−−−	−−−	−−−
	Vehicle failure	0.189	−0.048	−0.104	−0.037	−−−	−−−	−−−	−−−
	**Accident Consequence (Reference: Explosion)**								
	Fire	0.154	−0.001	−0.090	−0.031	0.135	−0.073	−0.030	−0.007
	Spill	0.164	−0.026	−0.156	−0.102	0.109	−0.098	−0.060	−0.020
	Non−spill	0.303	−0.002	−0.085	−0.027	0.178	−0.095	−0.034	−0.007
